# Alterations in performance and discriminating power of the death/suicide implicit association test across the lifespan

**DOI:** 10.1016/j.psychres.2024.115840

**Published:** 2024-03-04

**Authors:** Donna A. Ruch, Jeffrey A. Bridge, Jaclyn Tissue, Sean P. Madden, Hanga Galfavy, Marianne Gorlyn, Arielle H. Sheftall, Katalin Szanto, John G. Keilp

**Affiliations:** a The Abigail Wexner Research Institute at Nationwide Children’s Hospital Center for Suicide Prevention and Research, 444 Butterfly Gardens Dr., Columbus, OH 43215, United States; b Department of Pediatrics, The Ohio State University Wexner Medical Center College of Medicine, 370W. 9th Avenue, Columbus, OH 43210, United States; c Department of Psychiatry and Behavioral Health, The Ohio State University Wexner Medical Center, 370W. 9th Avenue, Columbus, OH 43210, United States; d Department of Psychiatry, Columbia University Vagelos College of Physicians and Surgeons, 1051, Riverside Drive, New York, NY 10032, United States; e Department of Biostatistics, Columbia University Medical Center, 1051 Riverside Drive, New York, NY 10032, United States; f Department of Psychiatry, University of Rochester Medical Center, 601 Elmwood Ave, Rochester, NY 14642, United States; g Department of Psychiatry, University of Pittsburgh, 3811 O’Hara St., Pittsburgh, PA 15213, United States

**Keywords:** Death/suicide implicit association, Suicide, Suicide risk assessment, Suicide prevention/early detection

## Abstract

The Death/Suicide Implicit Association Test (d/s-IAT) has differentiated individuals with prior and prospective suicide attempts in previous studies, however, age effects on test results remains to be explored. A three-site study compared performance on the d/s-IAT among participants aged 16–80 years with depression and prior suicide attempt (*n* = 82), with depression and no attempts (*n* = 80), and healthy controls (*n* = 86). Outcome measures included the standard difference (*D*) score, median reaction times, and error rates. Higher *D* scores represent a stronger association between death/suicide and self, while lower scores represent a stronger association between life and self. The *D* scores differed significantly among groups overall. Participants with depression exhibited higher scores compared to healthy controls, but there was no difference between participants with and without prior suicide attempts(*F*[2,242]=8.76, *p*<.001). Response times for participants with prior attempts differed significantly from other groups, with no significant differences in error rates. The *D* score was significantly affected by age (*β* =−0.007, *t* = 3.65, *p*<.001), with slowing of response times in older ages. Results suggest reaction time d/s-IAT *D* scores may not distinguish implicit thinking about suicide as response times slow with age, but slowed response times may be sensitive to suicide risk potentially indicating basic information processing deficits.

## 1. Introduction

Suicide is a major public health concern in the United States, accounting for over 45,000 deaths in 2020 (CDC, 2020). To support their vision for a “nation free from the tragic experience of suicide,” the National Action Alliance for Suicide Prevention (NAASP) established a research agenda to reduce the annual suicide rate 20 % by 2025 (NAASP, 2014). This agenda includes identifying improved capabilities to better detect individuals at highest risk for suicide across care settings. An existing limitation in our ability to effectively assess suicide risk is the almost exclusive reliance on self-report (Franklin et al., 2017; Kleiman et al., 2017). This can be especially problematic due to the transient nature and potential reluctance of individuals to express suicidal thoughts and behaviors (Carter et al., 2017; Franklin et al., 2017; Nock and Banaji, 2007). Detecting implicit associations with death and suicide has been offered as a more objective marker of suicide risk that can provide a complementary approach to patient self-report (Glenn et al., 2017a, b; Millner et al., 2018; Ellis et al., 2016).

The most widely studied measure of these implicit associations is the Death/Suicide Implicit Association Test (d/s-IAT) (Barnes et al., 2017; Nock et al., 2010; Sohn et al., 2021). The d/s-IAT examines implicit self-identification with death versus life by evaluating the relative difference in response times when life vs. death/suicide related stimuli are associated with the self (Nock et al., 2010; Nock and Banaji, 2007). Prior research has demonstrated that individuals at risk for suicide respond relatively more quickly than non-suicidal individuals to stimuli associating “death/suicide” and “me”, than when “life” and “me” are paired (Barnes et al., 2017; Glenn et al., 2017a,b; Nock et al., 2010; Tello et al., 2020). In a study by Nock et al. (2010), adults presenting to a psychiatric emergency department following a suicide attempt had relatively faster implicit associations pairing the self with death/suicide compared to those with other psychiatric emergencies. These patients also had a six-fold increase in the risk for a suicide attempt during the 6-month follow-up period (Nock et al., 2010). Similar findings were obtained in veterans in a psychiatric inpatient setting (Barnes et al., 2017), with veterans expressing relatively faster associations between self and death (compared to self and life) twice as likely to attempt suicide within 6 months of the baseline assessment (Barnes et al., 2017).

Evidence regarding the d/s-IAT and suicide risk has been inconsistent. In a study by Rath et al. (2021), d/s-IAT scores were not predictive of suicide attempts in adult psychiatric inpatients above and beyond depression, prior suicide attempts, and suicidal ideation. Glenn et al. (2019) found the d/-s-IAT was significantly associated with future suicide ideation in youth aged 12–19 years receiving psychiatric outpatient treatment, but not when controlling for baseline ideation. An additional study by Brent et al. (2021) among youth in pediatric emergency departments found the d/s-IAT to be only a modest predictor of future suicide attempts, with self-report of known suicidal risk factors showing greater predictive accuracy.

While research involving the d/s-IAT has been conducted with mostly younger populations, or populations within a relatively restricted age range (Barnes et al., 2017; Brent et al., 2021; Nock et al., 2010; Glenn et al., 2019), little is known about the discriminating power of the d/s-IAT in older adults. The current study examined whether implicit associations with death/suicide distinguished participants with depression and prior suicide attempts, participants with depression and no attempts, and healthy controls across the adult lifespan. Based on existing literature, we hypothesized that participants with prior suicide attempts would have higher difference scores on the d/s-IAT (faster relative association between self and death than to self and life) compared to participants with no attempts and healthy controls, and that participants with no attempts would have higher difference scores relative to healthy controls. We also sought to determine how age itself might affect the association between group status and d/s-IAT performance. The IAT outcome measure relies on reaction time differences between different conditions, and it is possible that age effects on reaction times may have an impact on the discriminating power of these scores. The present study examined participants aged 16 to 80 years, allowing for analysis of these aging effects, in addition to standard group differences.

## 2. Methods

### 2.1. Sample

Participants were recruited from three large metropolitan university hospitals and affiliated health care providers in Pittsburgh, PA, Columbus, OH, and New York City, NY as part of a collaborative multi-center study to examine age associations with clinical and neurocognitive risk factors for suicidal behavior. Exclusion criteria included a history of neurological disorder, delirium, psychosis, mania, dementia, or current substance abuse disorder. Individuals who scored <24 on the Mini-Mental State Examination (MMSE) as a test for cognitive impairment (Folstein et al., 1975) or who participated in electroconvulsive therapy within the past 6 months were also excluded from the study. The initial sample was comprised of participants aged 16 to 80 years (*N* = 309). There were 26 participants who did not complete the d/s-IAT and 35 participants who were excluded from analyses because d/s-IAT computer tasks did not meet established quality control criteria (Greenwald et al., 2003), leaving a total of 248 cases: individuals with depression and a history of suicide attempts (*n* = 82), individuals with depression and no history of suicide attempts (*n* = 80), and a comparison group with no prior suicidal ideation or behavior or any history of major psychiatric or neurologic illness (*n* = 86). Those excluded for failing quality control criteria were evenly distributed from among the clinical groups (χ^2^[2]=2.19, *p*=.335) and sites (χ^2^[2]=4.47, *p*=.107); however, they were on average older (t[281]=2.47, *p*=.014), less educated (t [281]=2.55, *p*=.011), and had lower estimated intelligence (t[274]= 4.60, *p*< .001).

Participants with depression met criteria for current major depressive disorder diagnosis based on the Structured Clinical Interview for DSM-IV Disorders (SCID) (First et al., 1995) and had a Hamilton Depression Rating Scale (HDRS-17) score >=14 (Hamilton, 1960). Suicide attempt was defined as any self-injurious behavior with stated or inferred intent to die (O’Carroll et al., 1996). Suicide attempt history was gathered using the Columbia Suicide History Form (CSHF) (Oquendo et al., 2008). The study was approved by the University of Pittsburgh Institutional Review Board, Abigail Wexner Research Institute at Nationwide Children’s Hospital Institutional Review Board, and New York State Psychiatric Institute Institutional Review Board. Written informed consent was obtained from all participants.

### 2.2. Assessments

Demographic characteristics and clinical history were obtained through structured interviews. The Vocabulary and Matrix Reasoning subtests of the Weschler Adult Intelligence Scale (4th edition) (Wechsler, 2008) were used to estimate overall intellectual ability and mental status was measured by the MMSE (Folstein et al., 1975). Hopelessness was assessed with the Beck Hopelessness Scale (BHS) (Beck et al., 1974) and impulsivity with the Barratt Impulsiveness Scale (Fossati et al., 2002; Barratt, 1965). Aggression was rated using the Buss-Perry Aggression Questionnaire (BPAQ) (Buss and Perry, 1992) and the Brown-Goodwin Assessment for Lifetime History of Aggression (Brown and Goodwin, 1986). The Hamilton Depression Rating Scale (HDRS) (Hamilton, 1960) was used as a clinician-rated measure of depression, and the Beck Depression Inventory (Beck and Steer, 1987) as a self-report of depression severity. The Personality Assessment Inventory–Borderline Features scale assessed attributes indicative of borderline personality disorder (Morey, 2014) and mental health disorders including substance abuse were determined via the SCID (First et al., 1995). Current and worst point suicide ideation was identified using the Scale for Suicide Ideation (SSI) (Beck et al., 1979). Suicide intent for the most lethal and most recent attempts among participants with a history of suicide attempts were assessed with the Beck Suicide Intent Scale (SIS) (Beck et al., 1974).

### 2.3. Death/Suicide implicit association test (d/s-IAT)

The d/s-IAT is a brief computer-administered test that assesses a participant’s tendency to implicitly associate death/suicide or life with self by measuring response times to stimuli representing the constructs of “Death/Suicide” and “Life” and the attributes of “Me” and “Not Me” (Nock et al., 2010). The version of the task used in this study contains 7 blocks of trials, with 3 single word practice blocks not used in scoring, and 4 paired word critical trial blocks: short (20 trials) and long (40 trials) blocks pairing “Me” words with words associated with life, and short and long blocks pairing “Me” words with words associated with death and suicide. Based on a standard IAT scoring algorithm (Greenwald et al., 2003), the calculated difference or *D* score is the difference in reaction times (as a function of pooled variability across conditions) when death/suicide words are paired with the self vs. others, and is conceptualized as the strength of each participant’s implicit association with death/suicide and themselves. Higher *D* scores represent a stronger association between death/suicide and self (i.e., relatively faster responding in the two “Death, Suicide”/“Me” blocks relative to the two “Life”/“Me” blocks), while lower scores represent a stronger association between life and self (Nock et al., 2010).

In addition to this standard outcome metric, median reaction times within each of the four critical conditions of the task were extracted, given the wide age range of the sample and the likelihood of age effects on response times and possible effects on the *D* score. A composite response time score was then computed as the overall mean of these four medians. Percent error scores for initial inaccurate associations within each condition and across all conditions were also computed, given their potential effect on response times that comprise the *D* score as well as the possible effects of age on these error scores.

### 2.4. Statistical analysis

Demographic and clinical characteristics were compared across groups using analysis of variance (ANOVA), chi-square (χ^2^), and exact tests as appropriate, and pairwise post-hoc comparisons. For each outcome measure (d/s-IAT *D* score, median reaction times within each condition, a composite response time, and error scores), an initial analysis of covariance (ANCOVA) was computed to examine group differences with age and study site as primary covariates. Demographic/clinical variables that differentiated groups were subsequently tested to evaluate their effect on outcome measures.

This was followed by a regression analysis examining the age by group interaction on each primary outcome (in addition to main effects of group, site, and age) to determine if age effects differed within each of the groups. To address potential problems of collinearity in this interaction model, and to aid in the interpretation of main and interaction effects, age was first centered with values reflecting the distance from the mean. Finally, correlations between each primary outcome measure and age, and between each outcome measure and other clinical variables were computed. All statistical tests were two-tailed, with an alpha level of 0.05. Statistical analyses were conducted with SPSS, version 28.0 (IBM SPSS Statistics, Somers, N.Y.) and STATA/IC version 16.0 (StataCorp, College Station, TX). Power analysis suggests that, with a moderate effect size (consistent with Millner et al., 2018 for the d/s-IAT in lifetime suicide attempters with approximately the same mean age as the sample here), sample sizes in this study are adequate for detecting a main effect for group with over 90 % power. An age x group interaction effect with a minimal to moderate effect size can be detected with 80 % power.

## 3. Results

### 3.1. Demographics and clinical ratings

The study sample was predominantly female, White, and non-Hispanic, with an overall average age of 41.8 ± 17.4 (range from 16 to 80) with no differences among the clinical groups. Years of education (*F*[2245]=6.31, *p*=.002), and MMSE scores (*F*[2245]=3.63, *p*=.028) were significantly lower among participants with a history of suicide attempts than healthy controls, but not compared to those with no attempts (Table 1). Estimated intellectual ability was comparable across groups, and within one point of the ceiling score for the test. Race differed significantly across groups (χ^2^[8]=29.93, *p*=.003), driven by significant differences among Multiracial and White participants. The percentage of Hispanic individuals was significantly higher among participants with prior attempts (χ^2^[2]=11.20, *p*=.004).

Both groups with depression had significantly higher depression severity and impulsivity scores compared to healthy controls, but did not differ from each other. Buss-Perry and Brown-Goodwin aggression scores (*F* [2245]=49.70, *p*<.001; *F*[2245]=23.95, *p*<.001), PAI Borderline Scale scores (*F* [2245]=190.08, *p*<.001), and Beck Hopelessness Scale scores (*F*(2245)=104.72, *p*<.001) were significantly higher in participants with prior suicide attempts compared to both other groups. Both patient groups had a high rate of comorbidity, with the majority of secondary diagnoses being anxiety disorders (Table 1). However, rate and type of comorbidities did not differ between groups.

The intensity of current (*U* = 1778.00, *z*= −5.05, *p*<.001) and worst point suicidal ideation (*U* = 853.50, *z*= −8.03, *p*<.001) was significantly greater in participants with prior suicide attempts than those with no attempts. Individuals with a history of suicide attempt made an average 2.4 ± 2.2 prior suicide attempts, with a maximum Beck Lethality score of 2.8 ± 2.2 (67.1 % lower lethality), and lethality of most recent attempt of 2.4 ± 2.1 (73.2 % lower lethality). Suicide Intent Scale score for the most lethal attempt was 16.3 ± 4.9, for the most recent attempt 16.1 ± 5.2.

### 3.2. Implicit association test d score

The d/s-IAT *D* score was initially compared across groups controlling for the effects of age, and site. The d/s-IAT *D* score differed significantly among the groups (*F*[2242]=8.76, *p*<.001), with scores in participants with depression significantly higher than those of the healthy comparison group, but not different between those with and without prior suicide attempts. Age was the only significant covariate (*F*[1242]= 33.72, *p*<.001).Demographic differences among the groups led to the additional inclusion of race, ethnicity, and years of education as covariates in the final model (Table 2). No additional variables were significantly associated with the d/s-IAT *D* score, while group (*F*[2237]=8.56, *p*<.001) and age (*F*[1237]=32.75, *p*<.001) effects were maintained.

**Fig. 1.**
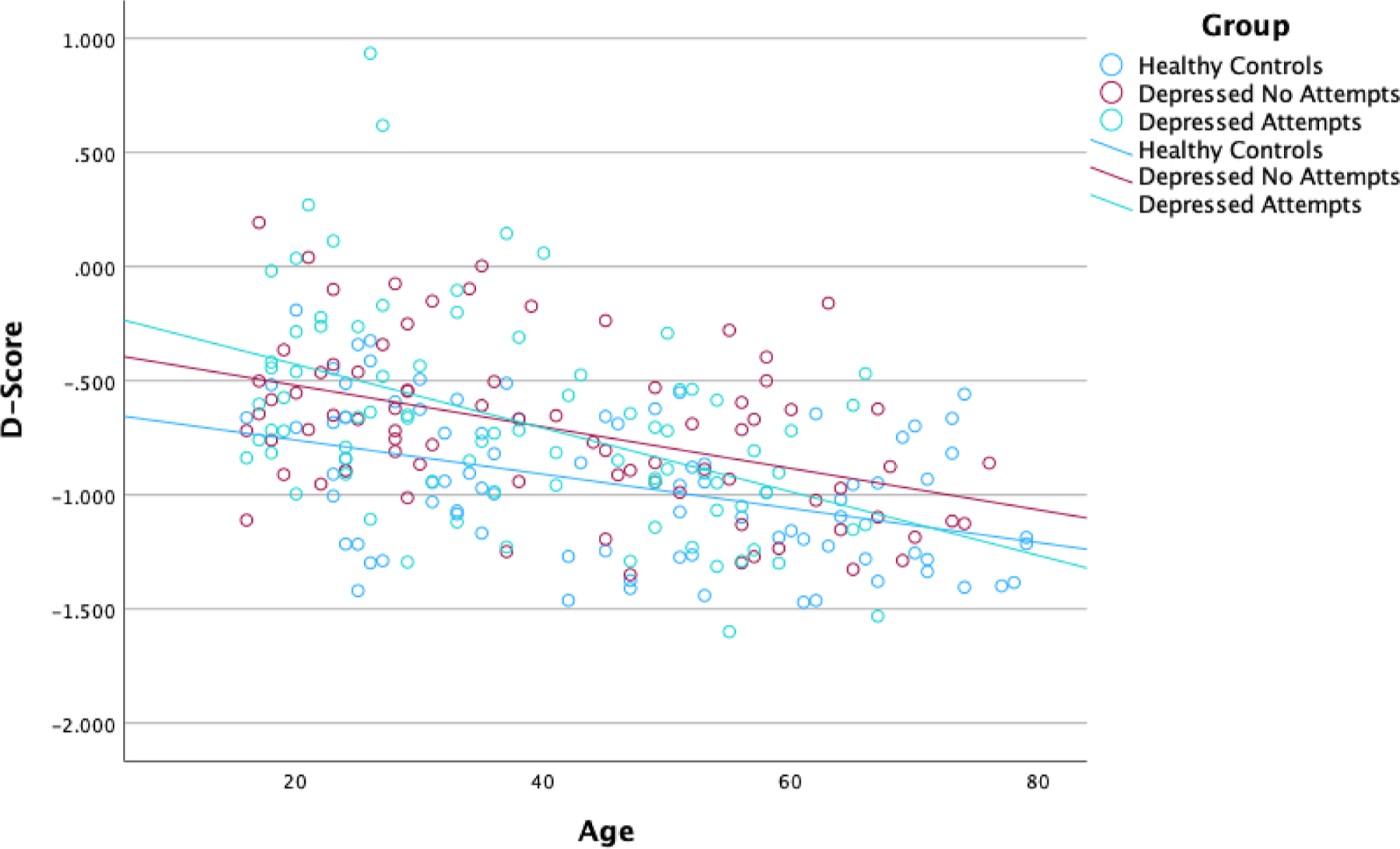
Implicit association test *D* score across the lifespan.

In the regression analysis with d/s-IAT *D* score as a dependent variable, including group, age, and the interaction of group by age as predictors, the overall model was significant (*F*[6241]=15.64, *p*<.001, R^2^=0.280), and the age (centered) main effect was significant across all groups (*β* = −0.007, *t* = 3.65, *p*<.001). The interaction effect was not significant for the pairwise comparison of participants with no attempts and healthy controls (*β* = −0.002, *t* = 0.58, *p*=.563), but was significant for the comparison of age effects in participants with suicide attempts relative to healthy controls (*β* = −0.006, *t* = 2.02, *p*=.044). As illustrated in Fig. 1, the *D* score declined more rapidly with age in participants with prior suicide attempters compared to healthy controls.

Within each of the groups, the correlation between age and *D* score was significant (*r*= −0.434, *p*<.001 in healthy controls, *r*= −0.445, *p*<.001 in participants with no attempts, and *r*= −0.465, *p*<.001 in participants with prior attempts). Almost no significant correlations were found between d/s-IAT *D* scores and known risk factors for suicide among participants with depression, except for a weak positive association with the Barratt Impulsiveness Scale score (*r* = 0.178, *p*=.027). Correlation with current suicide ideation was not significant (*r* = 0.03, *p*=.735).

### 3.3. Implicit association test median response times

In group comparisons of median response times within each condition of the d/s-IAT, participants with prior attempts exhibited the slowest response times in all blocks when adjusted for age (for short Life/Me block: (*F*[2242]=7.00, *p*<.001); long Life/Me block: (*F*[2242]=7.44, *p*<.001); short Death, Suicide/Me block: (*F*[2242]=4.65, *p*=.010); long Death, Suicide/Me block: (*F*[2242]=6.05, *p*=.003). Age was a highly significant covariate within each block (for short Life/Me block: *F* [1242]=19.71, *p*<.001; long Life/Me block: F[1242]=26.29, *p*<.001; short Death, Suicide/Me block: *F*[1242]=92.36, *p*<.001; long Death, Suicide/Me block: *F*[1242]=106.95, *p*<.001). For the composite response time measure, participants with prior suicide attempts exhibited the highest (slowest) overall reaction time of all groups (*F* [2242] = 7.92, *p*<.001). Once again, age was a highly significant covariate in these models (*F* [1242] = 98.66, *p*<.001). The addition of demographic covariates for race, ethnicity, and education did not alter these effects.

**Fig. 2.**
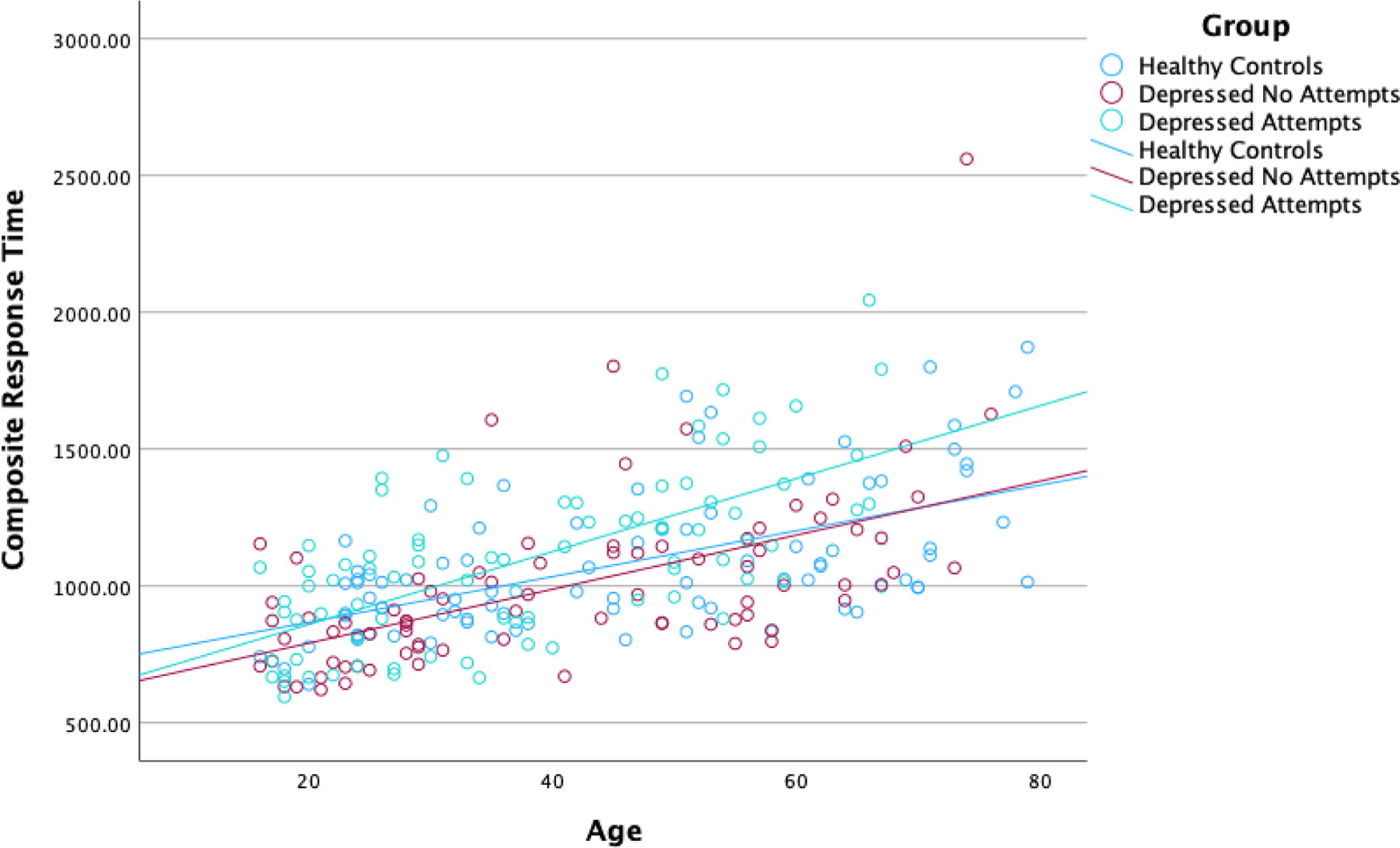
Implicit association test composite response times across the lifespan.

In the regression analysis on the composite response time measure, the overall model was again significant (*F*[6241]=25.28, *p*<.001, R^2^=0.386). Response times increased with age across all sites (*β* = 8.21, *t* = 5.99, *p*<.001). The interaction of age and group was not significant between participants with no attempts and healthy controls (*β* = 1.65, *t* = 0.81, *p*=.418), but was significant between participants with prior attempts and healthy controls (*β* = 4.99, *t* = 2.29, *p*=.023). The composite response time for participants with prior suicide attempts increased at a greater rate with age, and group differences appeared to increase at older ages (Fig. 2).

Correlations between age and the composite response time measure were significant in all groups (*r* = 0.587, *p*<.001 in healthy controls; *r* = 0.565, *p*<.001 in participants with no attempts; and *r* = 0.660, *p*<.001 in participants with prior attempts). Within the depressed sample, composite reaction time correlated with both the 16-item Hamilton Depression Rating Scale (*r* = 0.22, *p*<.001) and with current suicidal ideation (*r* = 0.31, *p*<.001). If we include both age and current suicidal ideation as covariates in a comparison of participants with past suicide attempts and those without attempts (healthy volunteers excluded due to lack of variability in suicidal ideation), both age (F[1155]=77.44, *p*< .001) and the current suicidal ideation score are significant covariates (F [1155]= 8.61, *p*=.004). However, the difference in reaction time between participants with attempts and those with no attempts remains significant (F[1155]= 4.78, *p*=.030). Within the sample of individuals with prior suicide attempts, composite reaction time correlated with suicide intent for both the maximum lethality attempt (*r* = 0.288, *p*=.009) and the most recent attempt (*r* = 0.294, *p*=.008).

### 3.4. Implicit association test error scores

Within each of the critical blocks of the task (Table 2), error percent did not differ across groups (for short Life/Me block: F[2242]=0.14, *p*=.872; long Life/Me block: F[2242]=0.54, *p*=.582; short Death, Suicide/Me block: F[2242]=0.20, *p*=.821; long Death, Suicide/Me block: F [2242]=1.23, *p*=.293). Error percent across all conditions also did not differ by group (F[2242]=0.76, *p*=.469), and only age (F[1242]=7.48, *p*=.007) was a significant covariate. Addition of demographic covariates for race, ethnicity, and education did not alter these effects.

Error percent did differ significantly between Life/Me blocks and Death, Suicide/Me blocks (*F*[1242]=22.28, *p*<.001), but did not differ across the groups (group by block interaction: (*F*[2242]=0.51, *p*=.599). In the regression model examining the group x age interaction, this interaction was non-significant (*β* = −0.02, *t*= −0.69, *p*=.491). The overall error rate correlated negatively with age within each of the groups (*r*= −0.275, *p*=.010) in healthy controls, (*r*= −0.269, *p*=.016) in participants with no attempts, and (*r*= −0.304, *p*=.006) in participants with prior attempts. The error percent was correlated with both the *D* score (*r*= −0.151, *p*=.018) and the overall response time (*r*= −0.188, *p*=.003), but in a manner that did not affect overall group effects or interactions on these variables.

### 3.5. Exploratory analyses

A number of exploratory analyses were conducted post hoc, given the divergence of current findings from those expected. The correlations between age and the *D* score, and between age and composite response time, suggest that response time itself was likely to be negatively associated with the computed *D* score, indicating the *D* score not only declines with age, but with overall response times themselves. This was true within all groups, with correlations between *D* score and composite response time significant in healthy controls (*r*= −0.284, *p*=.008), participants with no attempts (*r*= −0.295, *p*=.008), and participants with prior attempts (*r*= −0.389, *p*<.001).

The most extreme *D* scores (above zero) indicating faster reaction times in the death, suicide/me blocks – and hypothesized to be associated with the strongest implicit association between the self and death - occurred more frequently in participants with prior suicide attempts below the age of 40 (see Fig. 1, points above zero on the y-axis). Within this restricted age group, the difference in the proportion of participants with *D* scores above zero was significant (χ^2^[2]=6.72, *p*=.035), with no healthy controls (*n* = 0 of 39) in this range, 7.3 % of participants with no attempts (*n* = 3 of 41), and 15.2 % of participants with prior attempts (*n* = 7 of 46).

Previous research also suggests that *D* scores are sensitive to the recency of attempt (Glenn et al., 2017b; Nock et al., 2010). While there was no correlation between *D* scores and time since most recent attempt (*r* = 0.024, *p*=.831), and time since attempt did not differ across the sample of past suicide attempters. Analyses to identify a cutoff in both age and recency of attempt found that participants with prior suicide attempts under the age of 30 made their most recent attempt in the last six months had significantly higher *D* scores than all other groups (F[2, 62]=4.29, *p*=.018; mean *D* score of −0.746 ± 0.343 in healthy controls, −0.574 ± 0.307 in participants with no attempts, and −0.342 ± 0.489 in participants with prior attempts). If the age range was expanded to above age 30 and the time since most recent attempt extended beyond 6 months, participants with prior attempts were no longer significantly different than those with no attempts.

## 4. Discussion

Previous studies have demonstrated the predictive value of the d/s-IAT for suicide risk (Barnes et al., 2016; Ellis et al., 2016; Nock et al., 2010), but age effects appear to impact its discriminating power. In this cross-sectional adult lifespan sample, age was found to have a substantial negative association with the magnitude of the d/s-IAT *D* score. Our findings indicate that *D* scores no longer identified those with a history of suicide attempt in middle-aged and older participants. The difference in the age effect was small, but may reduce the potential predictive power of the IAT in older individuals. The *D* score was found to decline in conjunction with age-related increases in overall response times, suggesting that - psychometrically - it is not sufficiently independent of a general slowing of response times. However, differences in the *D* score between participants with depression (regardless of suicide attempt status) and healthy controls were found across the full adult age span.

In contrast to the *D* score, participants with a history of suicide attempts in this study had consistently slower overall response times relative to all other groups irrespective of age. These differences were not due to a greater likelihood of response errors (errors, in fact, were associated with faster overall response times). This slowing of response times in participants with prior attempts may represent an overall information processing deficit associated with risk for suicidal behavior. Slowing of response times has typically been associated with depression itself (Keilp et al., 2018) but not with suicide attempt status (Keilp et al., 2013; Richard-Devantoy et al., 2013, 2014). Previous research, however, has not examined an age range as wide as in the present study, and response time differences between participants with prior suicide attempts and other groups appeared to increase at older ages. Moreover, overall reaction times on the d/s IAT (and not the *D* score) were associated with the severity of current suicide ideation, suggesting a relationship to a core feature of suicidal behavior risk. In older-aged samples, the overall reaction time may be valuable for identifying risk in combination with other psychopathological factors.

In exploratory analyses, the *D* score identified the most extreme scorers as participants with a history of attempts under age 40 years. In addition, as a group, participants with prior suicide attempts had the highest *D* scores among those with very recent attempts within the last six months and under age 30 years. These results are consistent with existing literature which found significantly higher *D* scores in samples of participants with prior suicide attempts with mean ages below 30 years (Glenn et al., 2017b; Brent et al., 2021; Nock et al. 2007) and in participants with prior attempts where the most recent suicidal behavior was close in time to the assessment (Glenn et al., 2017b; Nock et al., 2010).

The current study highlights potential limitations of the d/s-IAT’s ability to identify suicide risk across the adult lifespan. It is possible that thinking about death in general is more normative in older ages thus attitudes toward death change with increasing age. This may mean the conceptual underpinnings of the d/s-IAT and the meaning of the *D* score might be altered at older ages. Data here suggest, however, that psychometric considerations may also have had a significant impact on the current study’s findings. Computationally, the *D* score is a simple difference in reaction times adjusted for their pooled variability. In previous studies of younger adult and adolescent samples, this score may not have been associated with overall slowing of response times across all task conditions due to an attenuation of range. In our sample of participants aged 16 to 80 years the association between response time and the *D* score is significant. Similar findings were noted in a study examining age group differences in implicit social cognitions using the Implicit Association Test (IAT) where researchers highlighted the importance of considering age-related slowing in response times when interpreting IAT results (Hummer et al., 2002). Likewise, in a methodological assessment of the IAT, authors acknowledged age related differences in response times as an extraneous influence of IAT effects (Nosek et al., 2007). Alternative computational approaches may be needed to make this difference score more independent of base reaction time and or age. For example, one alternative *D* score computation was generated by O’Shea et al. (2021) using disaggregated *D* scores for “me” and “not me” separately. Results showed that reduced self-identifications were more effective at distinguishing participants with prior suicide attempts from those with no attempts, than other-identifications (O’Shea et al., 2021). Demographically stratified normative data may also be needed to adjust *D* scores based on developmental trends in the healthy general population.

## 5. Limitations

The cross-sectional design of this study limits conclusions regarding individual developmental trajectories on the d/s-IAT. Second, most of the sample was female, White and non-Hispanic. Future studies should include greater representation of males and other racial and ethnic groups. Third, a higher percentage of individuals with more recent suicidal behavior, within the last six months to a year, may have resulted in stronger group differences, consistent with existing research (Glenn et al., 2017b; Nock et al., 2010). Fourth, age trends may have been affected by some older participants failing quality control criteria for inclusion in analyses. Future studies may consider ergonomic modifications to the task (e.g. larger stimuli, brighter screen, larger response keys) that may improve performance consistency in older adults.

## 6. Conclusion

The d/s-IAT administered in this study did not show group differences in past suicidal behavior at middle and older ages, primarily because the *D* score was sensitive to the slowing of reaction times with increasing age. Effects consistent with prior research were found in younger participants in this study and those with more recent suicidal behavior, but not across the full age span sampled here. Alternative methods of computing the *D* score may be needed to reduce its susceptibility to overall slowing of response time. Normative data may be needed to adjust for age effects.

Response times differentiated individuals with a history of suicide attempts across the adult lifespan and may reflect a basic information processing deficit that contributes to the risk for suicidal behavior. Future studies should incorporate measures to differentiate more basic aspects of task performance from more complex or higher-order functioning to identify factors most closely associated with suicide risk, and aim to characterize mechanisms by which this risk is conferred.

## Figures and Tables

**Table 1:** Demographic and clinical characteristics by group.

	Healthy Controls^1^		Depressed No Past Attempts		Depressed Past Attempts		p-Value	
	*N* = 86		*N* = 80		*N* = 82			
Site Location (%)							.135	
New York, NY	26	30.2	18	22.5	31	37.8		
Columbus, OH	27	31.4	36	45.0	24	29.3		
Pittsburgh, PA	33	38.4	26	32.5	27	32.9		
Age (Mean, SD)	45.7	18.5	41.3	17.5	38.2	15.3	**.019**	HC>ATT
Sex (% Female)	55.0	64.0	56.0	70.0	52.0	63.4	.618	
Race (% Yes)							**.003**	
Asian	5	5.8	3	3.8	6	7.3		
Black	18	20.9	8	10.0	16	19.5		
White	60	69.8	62	77.5	41	51.2		HC,NON<ATT
Multiracial	0	0.0	4	5.0	12	14.6		HC<NON<ATT
Other^2^	3	3.5	3	3.8	7	8.5		
Ethnicity (Hispanic or Latino% Yes)	6	7.0	6	7.5	18	22.0	**.004**	HC,NON<ATT
Education (Years) (Mean, SD)	15.7	2.2	15.2	2.5	14.4	2.3	**.002**	HC>ATT
Estimated Intellectual Ability (Mean, SD)	11.8	2.2	12.2	2.2	11.5	2.8	.164	
Mental State Exam (MMSE)	29.5	0.9	29.3	0.9	29.1	0.9	**.028**	HC>ATT
Depression								
Hamilton Depression (HDRS-16)	1.9	2.3	17.3	4.8	18.3	5.7	**<0.001**	HC<NON,ATT
Beck Depression Total	2.0	3.2	24.2	9.7	26.9	12.0	**<0.001**	HC<NON,ATT
Personality Assessment Inventory-Borderline Features (PAI)	9.7	6.0	32.2	10.7	40.5	13.7	**<0.001**	HC<NON<ATT
Barratt Impulsivity Score	54.6	8.7	67.0	10.3	70.3	14.2	**<0.001**	HC<NON,ATT
Aggression Scales								
Buss Perry Aggression Questionnaire	50.8	14.5	65.1	16.7	77.0	18.9	**<0.001**	HC<NON<ATT
Brown-Goodwin Aggression History	13.3	3.3	16.8	4.7	17.7	5.1	**<0.001**	HC<NON,ATT
Beck Hopelessness Scale	1.2	1.5	9.6	6.1	11.9	6.1	**<0.001**	HC<NON<ATT
Suicide Ideation								
Current Ideation (SSI)	0.0	0.0	6.6	10.1	15.4	11.8	**<0.001**	HC<NON<ATT
Worst Point Ideation (SSI)	0.0	0.3	11.1	11.4	27.2	4.6	**<0.001**	HC<NON<ATT
Clinical Characteristics (Current,% Yes)								
Anxiety Disorder	–	–	50	62.50	55	67.07	.539	
Post-Traumatic Stress Disorder (PTSD)	–	–	12	15.00	20	24.39	.133	
Past Substance Use Disorder	–	–	23	28.75	29	35.37	.339	

1 HC - Healthy Controls; NON – Depressed No Attempts; ATT – Depressed Attempts.

2 Other race includes American Indian/Alaskan Native (*n* = 2); Hawaiian/Pacific Islander (*n* = 1); Unknown/Not reported (*n* = 10).

**Table 2:** Implicit association test performance.

	Healthy Controls^1^ (*N =* 86)		Depressed No Past Attempts (*N* = 80)		Depressed Past Attempts (*N* = 82)			
Characteristic	Mean	SD	Mean	SD	Mean	SD	p-Value	
Implicit Association Test *D* Score^2^	−0.95	0.32	−0.71	0.36	−0.68	0.46	**<0.001**	HC<NON,ATT
Median Response Times								
Block 3 - Short Life/Me Block	839.2	223.6	833.5	234.7	919.4	272.1	**.002**	HC,NON<ATT
Block 5 - Long Life/Me Block	741.0	139.5	740.1	162.2	797.1	183.7	**<0.001**	HC,NON<ATT
Block 7 - Short Death, Suicide/Me Block	1589.0	524.1	1396.1	646.0	1550.1	605.7	**.016**	NON<ATT
Block 9 - Long Death, Suicide/Me Block	1156.0	330.2	1032.5	313.6	1137.5	395.0	**.003**	HC,NON<ATT
Composite Response Time (Mean of Medians)	1081.3	263.2	1000.6	305.5	1101.0	308.1	**.001**	HC,NON<ATT
Error Rates (% error)								
Block 3 - Short Life/Me Block	3.0	4.9	3.1	4.8	3.7	5.9	.872	
Block 5 - Long Life/Me Block	5.0	5.2	6.1	6.0	6.5	6.7	.582	
Block 7 - Short Death, Suicide/Me Block	11.3	9.7	12.0	10.2	12.7	10.7	.821	
Block 9 - Long Death, Suicide/Me Block	6.4	6.6	7.6	7.5	9.0	8.5	.293	
Composite Error Rate	6.2	5.2	7.1	5.5	7.8	6.3	.854	

1 HC - Healthy Controls; NON –Depressed No Attempts; ATT – Depressed Attempts.

2 Higher score associated with death/suicide and self (faster responding to death/me relative to life/me).
